# Adenovirus Early Proteins and Host Sumoylation

**DOI:** 10.1128/mBio.01154-16

**Published:** 2016-09-20

**Authors:** Sook-Young Sohn, Patrick Hearing

**Affiliations:** Department of Molecular Genetics and Microbiology, School of Medicine, Stony Brook University, Stony Brook, New York, USA

## Abstract

The human adenovirus genome is transported into the nucleus, where viral gene transcription, viral DNA replication, and virion assembly take place. Posttranslational modifications by small ubiquitin-like modifiers (SUMOs) are implicated in the regulation of diverse cellular processes, particularly nuclear events. It is not surprising, therefore, that adenovirus modulates and utilizes the host sumoylation system. Adenovirus early proteins play an important role in establishing optimal host environments for virus replication within infected cells by stimulating the cell cycle and counteracting host antiviral defenses. Here, we review findings on the mechanisms and functional consequences of the interplay between human adenovirus early proteins and the host sumoylation system.

## Minireview

Adenovirus (Ad) is a nonenveloped, icosahedral particle containing a linear double-stranded DNA genome. Since the first isolation from human adenoids in 1953 (1), 70 human Ad types grouped into seven species (HAdV-A to HAdV-G) have been identified (2). In a recent human virome study, the Ad subgroup C was detected in ~72% of screened sera from 569 human donors across four continents (3). Ads infect a wide range of vertebrates. Human Ad infection is usually mild and self-limiting; however, Ad infection can cause serious disease in infants, the elderly, and immunocompromised patients (4). Studies of Ad biology have contributed to breakthrough discoveries, including mRNA splicing (5, 6) and antigen presentation to T cells (7). Ad has been extensively used as a vector system for gene delivery in cell culture, gene therapy, and vaccine development (8).

Ad early proteins, produced prior to viral DNA replication, modulate viral gene expression, direct viral genome replication, and regulate diverse cellular processes in order to optimize the host environment for viral replication (9). These proteins are also involved in Ad-mediated cell transformation (9). Ad utilizes a multipronged strategy to inhibit host antiviral responses. Ad early proteins may interrupt different steps in a single cellular process or inhibit the same cellular factor via different mechanisms. For example, the Ad linear DNA genome triggers a cellular DNA damage response which is detrimental for virus replication, and different Ad early proteins target host factors involved in this response. Specifically, the Mre11-Rad50-Nbs1 (MRN) DNA repair complex is targeted by the early proteins E1B 55-kDa protein (E1B-55K), E4-open reading frame 6 (E4-ORF6), and E4-ORF3 (10). The promyelocytic leukemia (PML) and p53 proteins are also regulated by multiple Ad early proteins (10–14). Cross talk among the regulatory early proteins is crucial for efficient virus replication.

During Ad infection, cellular and viral proteins undergo dynamic posttranslational modifications (PTMs). The sumoylation system, which is important to maintain cell homeostasis, conjugates small ubiquitin-like modifiers (SUMOs) to lysine residues in substrate proteins (15, 16). SUMOs predominantly localize in the nucleus, where the replication of most DNA viruses takes place. Five mammalian SUMO genes, *SUMO-1* to *SUMO-5*, have been identified (15–17). Three major mammalian SUMO proteins, SUMO-1, SUMO-2, and SUMO-3, have been studied extensively. SUMO-2 shares 97% amino acid homology with SUMO-3 (often referred as SUMO-2/3) and 50% homology with SUMO-1. SUMO-2/3 can form polymeric SUMO chains via conjugation at SUMO-2/3 lysine residue 11 (15, 16). The abundant population of free SUMO-2/3 in cells may serve as a pool for immediate responses to various cellular stresses (15, 16). Protein sumoylation affects diverse cellular processes, including the regulation of protein-protein interactions, subcellular localization, protein stability, and enzymatic activities.

SUMO and ubiquitin proteins are similar in their structures and conjugation processes. Sumoylation is carried out by a cascade of enzymatic steps (15, 16). SUMO precursors are cleaved by SUMO-specific proteases (SENPs) at their carboxy termini to expose a diglycine motif. The processed forms of SUMO are covalently linked at the C-terminal glycine residue to a catalytic cysteine in the heterodimeric E1 SUMO-activating enzyme SAE1/SAE2 by an ATP-dependent reaction. SUMOs are subsequently transferred to a catalytic cysteine residue of the E2 SUMO-conjugating enzyme Ubc9. SUMOs may then be conjugated to a lysine residue(s) in a substrate protein directly by Ubc9 or in collaboration with a SUMO E3 ligase. SENPs deconjugate SUMO proteins from substrates, and free SUMOs can be recycled. To date, none of the identified mammalian SUMO E3 ligases has been reported to transfer SUMO via a catalytic cysteine residue (15, 16).

**FIG 1:**
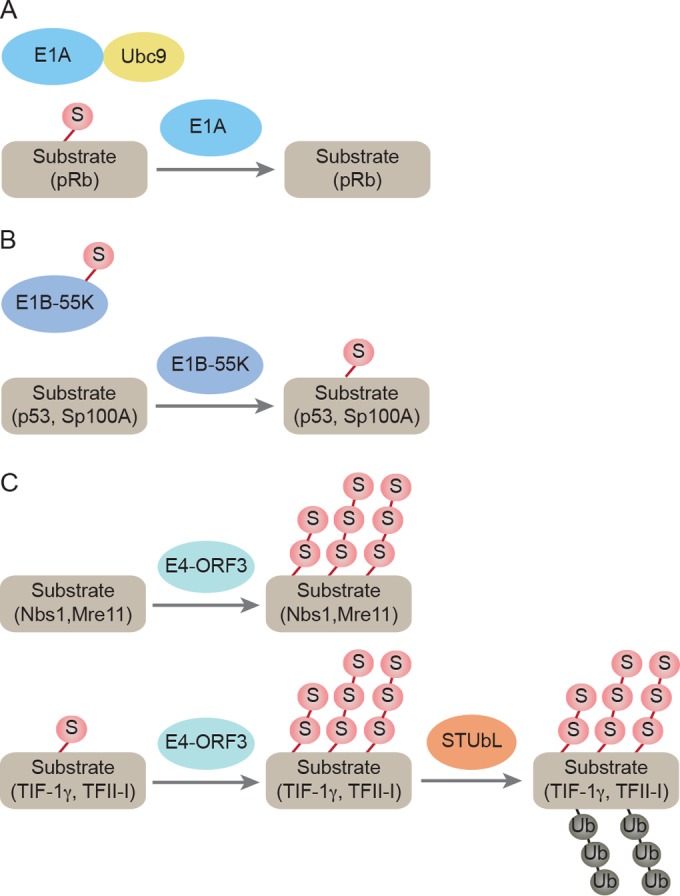
Schematic overview of the interplay between Ad early proteins and the host sumoylation system. (A) E1A directly binds to SUMO E2 enzyme Ubc9 and inhibits SUMO-1 conjugation to pRb. (B) E1B-55K is a SUMO substrate and an E3 ligase for p53 and Sp100A. (C) E4-ORF3 functions as a SUMO E3 ligase and an E4 elongase. E4-ORF3 triggers sumoylation of multiple host proteins and subsequent proteasomal degradation of some of the target proteins. S, SUMO.

In this review, we will focus on studies demonstrating the involvement of human Ad early proteins in the host sumoylation system. The E1A protein associates with the SUMO machinery and regulates sumoylation of target proteins. The E1B-55K protein is itself a SUMO substrate and an E3 ligase for p53 sumoylation. The E4-ORF3 protein induces sumoylation of multiple cellular proteins involved in a DNA damage response and functions as a SUMO E3 ligase and a SUMO E4 elongase (Fig. 1).

## THE E1A PROTEIN

E1A is the first gene that is transcribed during Ad infection, and it encodes two major phosphoproteins, termed 12S and 13S, alternatively referred to as the 243R and 289R protein, respectively (9, 18). These proteins share several conserved regions, CR1, CR2, and CR4, but only the 13S protein contains CR3. The E1A proteins interact with a large number of different cellular proteins to regulate a wide range of biological processes, including the transcription of viral and cellular genes, cell cycle, apoptosis, differentiation, transformation, and immune responses (9, 18).

In 1996, the first evidence for association between Ad and host sumoylation was obtained by screening of adenovirus 5 (Ad5) E1A protein binding partners using the yeast two-hybrid system (19). These results identified mouse Ubc9 as an E1A-interacting protein and further showed that CR2 of either Ad5 or Ad12 E1A is required for binding murine Ubc9 (mUbc9) in yeast (19). Fourteen years later, binding between E1A and human Ubc9 was confirmed in mammalian cells (20). Residues 118 to 123 (EVIDLT) within E1A CR2 were shown to be necessary and sufficient to bind the first alpha helix at the amino terminus of human Ubc9 using a yeast two-hybrid assay. The E1A T123H mutant (bearing a change of T to H at position 123), which is deficient in Ubc9 binding, localizes to both the cytoplasm and nucleus, whereas the wild-type E1A protein mainly localizes to the nucleus (20). E1A alters the physical properties of PML nuclear bodies (PML-NBs) such that they appear larger and fewer (21). Interestingly, the E1A T123H mutant has lost this ability (20). However, the interaction of E1A with Ubc9 is not required for oncogenic transformation of mouse embryonic fibroblasts (MEFs) and E1A expression does not affect global cellular protein sumoylation (20). The E1A protein was shown to suppress polysumoylation in yeast (20); further studies are required to examine the function of this E1A in mammalian cells.

The E1A protein also has been shown to inhibit the conjugation of SUMO-1 to the retinoblastoma protein (pRb). A pRb mutant that is deficient in binding to E1A exhibits enhanced sumoylation (22). To date, pRb is the only known protein whose sumoylation is regulated by E1A. It is not known whether the association between E1A and Ubc9 is required for inhibition of pRb sumoylation. Although the sumoylation-deficient pRb K720R mutant showed a slightly higher repressive effect on an E2F-responsive promoter than did wild-type pRb (22), the function of pRb sumoylation remains elusive. Multiple viral oncoproteins have been shown to regulate sumoylation of cellular pocket proteins, such as pRb, p107, and p130 (22, 23), and it will be interesting to study the role of SUMO modification on pocket protein activities. Since E1A does not affect global cellular protein sumoylation or the sumoylation of two known SUMO substrates (20), E1A most likely selectively targets specific cellular proteins to regulate their sumoylation.

## THE E1B-55K PROTEIN

The Ad E1B-55K protein is also multifunctional and required for Ad-mediated oncogenic transformation of primary cells in cooperation with E1A. It regulates the nuclear export of viral mRNAs, inhibits the nuclear export of cellular mRNAs, inhibits the transcriptional activation properties of p53, and induces the proteasomal degradation of specific cellular proteins (9, 24). As a consequence, E1B-55K regulates virus replication, viral and cellular gene expression, a DNA damage response, and host cell apoptosis.

E1B-55K directly binds to the N terminus of p53 and inhibits p53-mediated transcriptional activation (9, 24). E1B-55K also associates with other Ad early proteins, specifically, the E4-ORF3 and E4-ORF6 proteins. Together with E4-ORF6, Ad5 E1B-55K binds to an E3 ubiquitin ligase complex containing the cellular proteins elongins B and C, cullin 5 (Cul5), and RBX1 (25, 26). The E1B-55K–E4-ORF6 complex tethers the Cul5 complex to different cellular proteins in order to target them for ubiquitin conjugation and proteasomal degradation. Multiple substrate proteins of this viral E3 ubiquitin ligase complex have been identified, such as p53, integrin α3, the MRN complex components, DNA ligase IV, Bloom helicase (BLM), Tip60, and SPOC1 (26–32). Ad5 E1B-55K also targets the chromatin-remodeling factor Daxx (death-associated protein) for proteasomal degradation by an E4-ORF6-independent mechanism (33).

The subcellular localization pattern of E1B-55K is complex, heterogeneous, and significantly altered depending on the presence of other Ad early proteins. The E1B-55K protein alone predominantly localizes to large cytoplasmic aggregates in transfected cells (34–36). Upon wild-type Ad5 infection, E1B-55K exhibits a diffuse nuclear distribution overlaid with PML-NBs and small perinuclear aggregates. As virus replication progresses, E1B-55K moves to nuclear viral replication centers and accumulates at perinuclear aggresomes at the microtubule-organizing center (MTOC) (11, 37–41). E1B-55K contains a leucine-rich nuclear export signal (NES), which mediates chromosome region maintenance 1 (CRM-1)-dependent nuclear export (42). E1B-55K colocalizes in nuclear track-like (see “The E4-ORF3 Protein” below) structures formed by E4-ORF3 in E4-ORF6-deficient Ad-infected cells, and it is diffusely localized throughout the nuclei of E4-ORF3-deficient Ad-infected cells (11, 40). E1B-55K regulates multiple PTMs, including sumoylation and ubiquitination, and moreover, E1B-55K is itself a substrate for phosphorylation and sumoylation.

Ad5 E1B-55K is the only known SUMO substrate among Ad proteins. SUMO-1, SUMO-2, and SUMO-3 have been shown to be covalently attached to the E1B-55K lysine 104 residue at a consensus sumoylation motif (ψKXE/D, where ψ is a large hydrophobic residue and X is any residue) (34, 43). Lysine 104 is conserved among divergent human Ad serotypes. However, E1B-55K proteins from species A, F, and G lack the hydrophobic residue adjacent to the lysine within the consensus sumoylation motif (34, 43), and further studies are required to determine whether sumoylation of E1B-55K is serotype specific. Mutation of Ad5 E1B-55K lysine 104 to arginine (K104R) completely abolishes sumoylation (34). The E1B-55K K104R mutant is defective in the transformation of primary baby rat kidney (BRK) cells in cooperation with E1A and in the inhibition of p53 transactivation activity (34). By taking advantage of the sumoylation-deficient mutant, the physiological effects and biological properties of E1B-55K sumoylation have been studied intensively. Sumoylation of E1B-55K affects its interaction with partner proteins, subcellular localization, and ubiquitin E3 ligase activity. The interaction between E1B-55K and some cellular proteins is affected by the sumoylation status of E1B-55K. Without other viral proteins, E1B-55K selectively binds PML isoforms IV and V. The K104R mutant protein still can bind PML-IV, but not PML-V (35). The association of E1B-55K with a tumor suppressor, Sp100 (speckled protein, 100 kDA), is also impaired by the mutation in its sumoylation site (44). In contrast, the E1B-55K K104R mutant binds to p53, Mre11, and Daxx as efficiently as the wild-type protein and induces the degradation of p53 and Mre11 (34, 36, 37). However, this sumoylation-deficient mutant fails to target Daxx for proteasomal degradation (36). As described previously, the E4-ORF6 protein is required for Ad-induced degradation of p53 and Mre11 but not Daxx (33). These results imply that sumoylation of E1B-55K is not essential for binding to some cellular target proteins and E4-ORF6-dependent proteasomal degradation but that it is crucial for E4-ORF6-independent protein degradation.

Sumoylation has been shown to regulate the subcellular localization of substrate proteins. Distinct subcellular localization of the K104R mutant and wild-type E1B-55K was suggested as one of the reasons for the functional defect in sumoylation-deficient E1B-55K (34). In expression plasmid-transfected cells, wild-type E1B-55K and the K104R mutant protein localize to large cytoplasmic bodies and do not affect the distribution of endogenous SUMO-1. However, when CRM1-dependent nuclear export is blocked by leptomycin B (LMB) treatment, wild-type E1B-55K is localized in the nucleus and overlaid with SUMO proteins in PML-NBs, while the K104R mutant remains in the cytoplasm and fails to show any colocalization with SUMO proteins (34, 43). Inactivation of the E1B-55K NES within the K104R mutant protein still fails to induce PML-NB colocalization (45), indicating that E1B-55K sumoylation is required for its association with PML-NBs and nuclear accumulation (34, 45). Since the subcellular localization of E1B-55K is dramatically altered by the presence of E4-ORF3 and E4-ORF6 proteins (39, 40), the effect of sumoylation on E1B-55K localization has been determined in combination with these proteins (37). In Ad-infected cells, wild-type E1B-55K is predominantly localized at viral replication centers, as described above, while the K104R mutant protein is mostly accumulated as perinuclear aggregates (37). Interestingly, the K104R mutant protein is still localized to cytoplasmic aggregates in the absence of a functional NES and/or in the presence of LMB, suggesting that sumoylation plays a crucial role in targeting E1B-55K to viral replication centers and in the nucleocytoplasmic shuttling of this protein in a CRM1-independent pathway (37). Moreover, it has been shown that the sumoylation status of E1B-55K affects the subcellular localization of its target proteins. SUMO conjugation of E1B-55K is required for the retention of p53 at PML-NBs with E1B-55K and the subsequent nuclear export and inhibition of p53 (45). It is not well understood why the binding between E1B-55K and some of the partner proteins is independent of E1B-55K sumoylation, although SUMO conjugation of E1B-55K alters its subcellular localization remarkably.

Multiple factors regulating E1B-55K sumoylation have been identified, including its subcellular localization and phosphorylation status (37, 43) and viral and cellular proteins (39, 46). Sumoylation of E1B-55K affects its subcellular localization and vice versa. Enhanced nuclear retention of E1B-55K by abolishing a functional NES leads to a significant increase of its SUMO conjugation and subsequent increase of p53 transactivation activity and oncogenic transformation of primary cells (37, 43). Consistent with the findings of cross talk between protein phosphorylation and sumoylation, it has been shown that E1B-55K phosphorylation at C-terminal residues is required for its sumoylation (43). The phosphorylation-defective E1B-55K protein exhibits a drastic decrease in SUMO conjugation in either transfected or infected cells and subsequent loss of its functions (43). E1B-55K sumoylation is also regulated by another viral protein, E4-ORF6 (39). Sumoylated E1B-55K is more abundant in cells infected with an E4-ORF6-deficient virus than in cells infected with a wild-type virus (39). Since phosphorylation-defective E1B-55K displays increased nuclear staining but lack of relocalization into viral replication centers (43, 47) and E4-ORF6 also affects E1B-55K localization, as described above, the effect of the complicated correlation between E1B-55K PTMs and subcellular localization on E1B-55K functions needs to be further investigated. Recently, transcriptional intermediary factor 1β (TIF-1β) (also known as TRIM28, KAP1, and KRIP1) was shown to bind to E1B-55K and facilitate sumoylation of E1B-55K, whereas Ad infection decreases TIF-1β sumoylation by an unknown mechanism (46). It was suggested that Ad regulates TIF-1β sumoylation to minimize its epigenetic gene silencing properties and promote Ad replication (46).

The Ad5 E1B-55K protein is known as a SUMO E3 ligase, as well as a substrate. Interaction between Ad5 E1B-55K and the SUMO E2 enzyme Ubc9 has been shown by coimmunoprecipitation from cell extracts, but E1B-55K sumoylation is dispensable for this binding (43). The tumor suppressor p53 is the best characterized sumoylation target of E1B-55K. Sumoylation of p53 is induced by E1B-55K in mammalian cells and in an *in vitro* sumoylation system (45, 48). E1B-55K-mediated sumoylation of p53 relies on interaction with E1B-55K and sumoylation of E1B-55K, and it is required for E1B-55K-mediated inhibition of p53 transactivation (45, 48). E1B-55K also stimulates SUMO-2 conjugation to the PML-NB component Sp100A (44). E1B-55K sumoylation is required to induce Sp100A sumoylation and mediate the association of Sp100A and p53, their colocalization to PML-NBs, and repression of Sp100A-induced p53 activation (44). However, it is unclear whether E1B-55K directly functions as a SUMO E3 ligase for Sp100A.

As discussed above, E1B-55K associates with PML isoforms IV and V, and binding to PML-V depends on E1B-55K sumoylation (35). E1B-55K–PML binding is not required for the interaction of E1B-55K with p53, Mre11, or Daxx. However, E1B-55K mutants that are defective in binding PML fail to induce p53 sumoylation, repress p53-mediated transcriptional activation, and promote oncogenic transformation in conjunction with E1A (49). Thus, there is a complex and multifaceted effect of E1B-55K sumoylation that relates to different aspects of its function.

## THE E4-ORF3 PROTEIN

The E4-ORF3 protein self-assembles into higher-order protein multimers (50, 51) to form a unique filamentous network in the nucleus of Ad-infected cells that is referred to as tracks (12). E4-ORF3 generates distinct binding interfaces for different cellular target proteins (50) and recruits numerous proteins, including PML-NB components (12), the Mre11-Rad50-Nbs1 (MRN) complex (27, 52), SUMO proteins (53), TIF-1α (TRIM24) (54), TIF-1γ (also known as TRIM33 and Ectodermin) (55), and the general transcription factor TFII-I (56). This event causes sequestration of target proteins and enables E4-ORF3 to interfere with and exploit diverse cellular processes.

Ad5 E4-ORF3 promotes sumoylation of multiple cellular proteins in cultured cells (53, 56–58). These target proteins can be divided into two groups based on their sumoylation and proteasomal degradation patterns. The first group includes Mre11 and Nbs1. Mre11 and Nbs1 sumoylation is undetectable in the absence of E4-ORF3, and these proteins are transiently sumoylated following Ad5 infection, before their protein levels are decreased in infected cells due to E1B-55K/E4-ORF6-mediated proteasomal degradation (53). In the absence of E1B-55K/E4-ORF6, E4-ORF3 still induces transient Mre11 and Nbs1 sumoylation. The second group includes TFII-I and TIF-1γ. Their sumoylation is detectable in the absence of E4-ORF3 but is increased in the presence of E4-ORF3, and SUMO conjugation persists as long as the proteins persist (57, 58). These substrates are targeted for proteasomal degradation through an E4-ORF3-dependent, E1B-55K/E4-ORF6-independent pathway (57–59).

The correlation between the subcellular localization and sumoylation of E4-ORF3 target proteins has been investigated. Mre11 and Nbs1 desumoylation was enhanced by SENP1 overexpression, but both proteins were still relocalized by E4-ORF3, suggesting that sumoylation is not required for relocalization of target proteins into nuclear tracks (53). Instead, relocalization of target proteins into E4-ORF3 nuclear tracks is a prerequisite for substrate sumoylation and proteasomal degradation (53, 56–58). An Ad5 E4-ORF3 mutant harboring the separation-of-function mutations D105A and L106A forms tracks and relocalizes SUMOs, PML, and TIF-1γ but not the MRN complex and TFII-I (52, 53, 57, 58). This mutant protein induces sumoylation of TIF-1γ but not Mre11, Nbs1, and TFII-I (53, 57, 58). During Ad5 infection, SUMO-2/3 undergoes dynamic changes in subcellular distribution. As soon as E4-ORF3 tracks are formed, SUMO-2/3 is recruited to the tracks and then relocalized into viral replication centers at later times of Ad infection (53, 60). Nbs1 also shows the same localization patterns as SUMO-2/3 during Ad infection, while TFII-I and TIF-1γ remain in E4-ORF3 tracks until later times of infection, when they are degraded. It is unclear why Mre11 and Nbs1 are transiently sumoylated by E4-ORF3, whereas the sumoylation of TFII-I and TIF-1γ persists during Ad infection.

Recently, direct evidence that the E4-ORF3 protein functions as a SUMO E3 ligase has been revealed (58). Recombinant Ad5 E4-ORF3 protein promotes SUMO-3 conjugation to TIF-1γ in a reconstituted *in vitro* reaction system. In the absence of substrates, E4-ORF3 intensively facilitates poly-SUMO assembly, demonstrating that E4-ORF3 functions as a SUMO E4 elongase (58). Interestingly, a multimerization-deficient E4-ORF3 mutant (L103A) protein has significantly impaired activities compared to those of the wild-type protein in *in vitro* reactions. Using polymerization-deficient SUMO-3, E4-ORF3 was shown to enhance SUMO-3 conjugation on TIF-1γ, as well as poly-SUMO chain elongation on acceptor SUMO-3 (58). Since the discovery of SUMO-targeted ubiquitin ligases (STUbLs) that bind poly-SUMO chains to target polysumoylated proteins for ubiquitination-dependent proteasomal degradation, polysumoylation has emerged as an important mechanism connecting sumoylation and ubiquitination (61). Two human STUbLs, RNF4 and RNF111, and three viral STUbLs, ORF61 (varicella zoster virus), ICP0 (herpes simplex virus), and k-Rta (Kaposi’s sarcoma-associated herpesvirus), have been identified (62–67). The recombinant E4-ORF3 protein neither binds poly-SUMO chains nor exhibits any STUbL activity *in vitro* (58). It seems likely that E4-ORF3 may recruit a cellular STUbL to selectively target some E4-ORF3 SUMO substrates, such as TFII-I and TIF-1γ, for proteasomal degradation.

Since the target proteins of E4-ORF3 exhibit distinct sumoylation patterns (56), it is possible that sumoylation of some substrates is achieved directly by E4-ORF3 E3 SUMO ligase activity, while other substrates may require a cellular SUMO E3 ligase. One of the known SUMO E3 ligases, PIAS3, has been shown to relocalize into the E4-ORF3 tracks in transient expression assays (60), but the role of PIAS3 during Ad infection and on E4-ORF3-mediated sumoylation is not known.

## CONCLUSIONS

Viruses interplay with the host sumoylation system to manipulate a variety of cellular responses. Ad early proteins can become a target of sumoylation, interact with the SUMO machinery, and regulate sumoylation of cellular proteins (Fig. 1). The Ad E1A protein binds Ubc9 and inhibits sumoylation of pRb, presumably to interfere with pRb and stimulate E2F activity. E1A also alters the physical properties of PML-NBs through a mechanism that may involve sumoylation. Further studies will be required to identify and characterize other cellular proteins whose SUMO modification is regulated by E1A and investigate a molecular mechanism of sumoylation inhibition mediated by E1A. The Ad5 E1B-55K protein is both a SUMO substrate and a SUMO E3 ligase. Sumoylation of the Ad5 E1B-55K protein regulates its subcellular localization and interaction with cellular binding partners and enhances its functional properties. p53 is the best characterized target of E1B-55K SUMO E3 ligase activity. p53 sumoylation is required for E1B-55K binding and sumoylation and for E1B-55K-mediated inhibition of p53 transactivation. p53 is negatively regulated by multiple mechanisms in Ad-infected cells, underscoring the critical role that p53 plays in the inhibition of Ad replication. The Ad E4-ORF3 protein induces the sumoylation of cellular proteins, many of which are involved in a DNA damage response and, in some cases, subsequent proteasomal degradation. E4-ORF3 functions as a SUMO E3 ligase for TIF-1γ and, likely, other cellular proteins and as a SUMO E4 elongase. Increased sumoylation of cellular proteins by Ad early gene products is generally associated with inhibition of their antiviral activities. Most of the studies involving Ad regulation of cellular protein sumoylation have utilized adenovirus type 5. It will be interesting to determine how other Ad serotypes impact host sumoylation and how this pertains to the properties of cell types associated with different Ad infections. Not surprisingly, with each example of Ad regulation of the host sumoylation machinery, this system is exploited to augment the lytic virus life cycle. How the host sumoylation system may be altered in the context of latent adenovirus infection has not been investigated.

An enigma in the SUMO field has been how sumoylation affects the overall function of a protein when, typically, only a small percentage of the population of that protein is sumoylated (68). Several ideas have been proposed to explain this conundrum, including the idea that protein sumoylation may be required to initiate but not maintain a regulatory event (for example, repression of a transcription factor) or that sumoylation is only needed transiently to initiate subsequent processes (68). The studies by Pennella et al. pertaining to E1B-55K and p53 sumoylation support a different model (45). E1B-55K and p53 oligomerize themselves and with each other to form a molecular network within cells. Only a small fraction of each of these proteins is sumoylated. This model proposes that once a network of interacting proteins has formed, interactions between sumoylated proteins within the network and other proteins with SIMs (SUMO interaction motifs) (for example PML) could have broad effects on the properties of the overall network. Taking PML as an example, sumoylation of a small fraction of E1B-55K and p53 would move the E1B-55K:p53 protein network to PML-NBs. All three of these models may be correct and pertain to specific examples of protein sumoylation.
